# Emulating real-world GLP-1 efficacy in type 2 diabetes through causal learning and virtual patients

**DOI:** 10.1371/journal.pdig.0000927

**Published:** 2025-07-21

**Authors:** Calum Robert MacLellan, Hristo Petkov, Conor McKeag, Feng Dong, David John Lowe, Roma Maguire, Sotiris Moschoyiannis, Jo Armes, Simon Skene, Alastair Finlinson, Christopher Sainsbury

**Affiliations:** 1 Department of Biomedical Engineering, University of Strathclyde, Glasgow, United Kingdom; 2 Department of Computer and Information Sciences, University of Strathclyde, Glasgow, United Kingdom; 3 School of Cardiovascular & Metabolic Health, University of Glasgow NHS Greater Glasgow and Clyde, Glasgow, United Kingdom; 4 Digital Health Validation Lab, University of Glasgow, Glasgow, United Kingdom; 5 Emergency Department, Queen Elizabeth University Hospital, Glasgow, United Kingdom; 6 School of Computer Science and Electronic Engineering, University of Surrey, Guildford, United Kingdom; 7 School of Data Science and Computer Science, Shandong Women's University, Jinan, Shandong, China,; 8 Faculty of Health and Medical Sciences, University of Surrey, Guildford, United Kingdom,; 9 Surrey Clinical Trials Unit, University of Surrey, Guildford, United Kingdom,; 10 Institute of Applied Health Research, University of Birmingham, United Kingdom; University of Exeter, UNITED KINGDOM OF GREAT BRITAIN AND NORTHERN IRELAND

## Abstract

Randomized controlled trials (RCTs) remain the benchmark for assessing treatment effects but are limited to phenotypically narrow populations by design. We introduce a novel generative artificial intelligence (AI) driven emulation method that infers effect size through *virtual* clinical trials, which can emulate the RCT process and potentially extrapolate into wider populations. We validate the virtual trials by comparing the predicted impact of glucagon-like peptide-1 (GLP-1) agonists on HbA1c in type-2 diabetes (T2DM) with its true efficacy established in the LEAD-5 trial. Our emulation model learns treatment effects from real-world evidence data by a combined generative AI and causal learning approach. Training data comprised pre- and post-treatment outcomes for 5,476 people with T2DM. We considered three treatment arms: GLP-1 (Liraglutide), basal insulin (glargine), and placebo. After training, virtual trials were conducted by sampling 232 virtual patients per arm (according to the LEAD-5 inclusion criteria) and predicting post-treatment outcomes. We used difference-in-differences (DiD) for pairwise comparisons between arms. Our goal was to emulate LEAD-5 by demonstrating a significant DiD in post-treatment HbA1c reduction for GLP-1 compared to basal insulin and placebo. We found significant differences in HbA1c reduction for GLP-1 vs basal insulin (-1.21 mmol/mol (-0.11%); p < 0.001) and GLP-1 vs placebo (-2.58 mmol/mol (-0.24%); p < 0.001) in our virtual populations, consistent with LEAD-5 (Liraglutide vs glargine: -2.62mmol/mol (-0.24%); p = 0.0015, Liraglutide vs placebo: -11.91 mmol/mol (-1.09%); p < 0.0001). The causal AI-powered clinical trials can emulate LEAD-5 in important measurements for T2DM. Our algorithm is specialty agnostic and can explore counterfactual questions, making it suitable for further study in the generalizability of RCT results in real-world populations to support clinical decision-making and policy recommendations.

## Author summary

Clinical trials are the gold standard for evaluating how well treatments work, but they have limitations. These trials typically include a very specific group of patients, meaning their findings may not fully apply to the broader population. They are also expensive and time-consuming. To address these challenges, we developed an artificial intelligence-based approach that can simulate clinical trials using real-world data. Our model generates virtual patient populations that reflect real-world diversity, allowing us to estimate how treatments like GLP-1 receptor agonists impact blood sugar levels in people with Type 2 diabetes. By using a combination of causal discovery and machine learning, our approach mimics the process of a traditional clinical trial while adjusting for key factors that influence treatment outcomes. We tested our method by replicating the results of a well-known diabetes trial (LEAD-5) and found that our virtual trial produced similar findings. This suggests that the causal AI-powered trial emulation could help extend clinical trial results to a wider range of patients, supporting more personalized treatment decisions and improving healthcare policy. Our approach could be applied to other diseases, providing a powerful tool for medical research beyond diabetes.

## Introduction

Randomized controlled trials (RCTs) are accepted as the gold standard for assessing the effect size of therapeutic agents on an outcome of clinical interest [1–4]. By design, they implement specific inclusion and exclusion criteria, intentionally limiting the phenotypic range of participants involved. However, the real-world patient population is phenotypically diverse and frequently presents with multiple complications and other existing co-morbidities, which are often under-represented in RCT cohorts. This discrepancy between the controlled environment of RCTs and the more diverse real-world scenarios raises questions about whether effect sizes observed in practice defining RCTs are truly applicable to patients encountered in routine clinical practice. Moreover, RCTs are resource intensive: forming intervention groups spanning a broad range of therapies, each with diverse populations, and sufficiently sized to carry statistical significance, becomes increasingly challenging as the study scale grows. Together, both factors in RCTs — deviating from real-world populations and scalability — limit the extent to which we can confidently make causal statements about the effect size in real-world clinical practice.

Real-World Evidence (RWE) studies using clinical practice data present an opportunity to investigate effect size across phenotypes that extend beyond those explored within an RCT, given appropriate methodologies [5–7]. The importance of real-world data and digital trial methods in regulatory decision-making is increasingly recognised by international agencies. The U.S. Food and Drug Administration (FDA), under the 21st Century Cures Act, has established a formal framework for using RWE to support drug approval, indication expansion, and post-market surveillance, particularly in settings where randomized controlled trials (RCTs) are infeasible or incomplete [15]. Similarly, the European Medicines Agency (EMA) has issued Good Clinical Practice guidelines for the use of computerised systems and artificial intelligence in clinical trials, including expectations around data governance, electronic data capture, and algorithm validation [16].

However, RWE databases are inherently observational, meaning any attempt to emulate a target trial with RWE data is still restricted to the specific eligibility criteria of the trial of interest. In other words, although we can interpret treatment effects from real-world data, we have a limited capacity to directly emulate interventions and confirm a wide range of new hypotheses that lie outside an RCTs remit.

**Fig 1:**
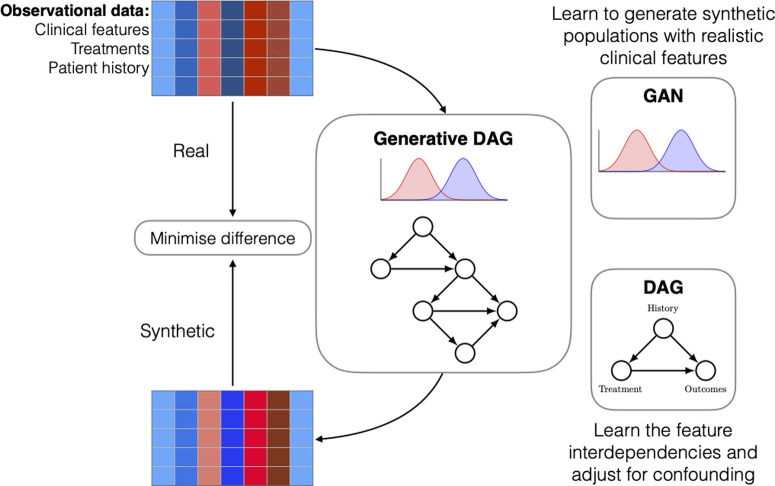
Workflow of the proposed trial emulation model: The model learns treatment effects from a real-world observational dataset by improving its data reconstruction quality through a distance minimization between the real and synthetic (virtual) data. Synthetic samples are drawn from a generative directed acyclic graph (DAG) that jointly models the marginal distributions (GAN) and causal relationships amongst its features. Confounding refers to variables that influence both treatment assignment and outcomes (e.g., age, BMI, prior treatments), and are adjusted for in the causal graph.

**Fig 2:**
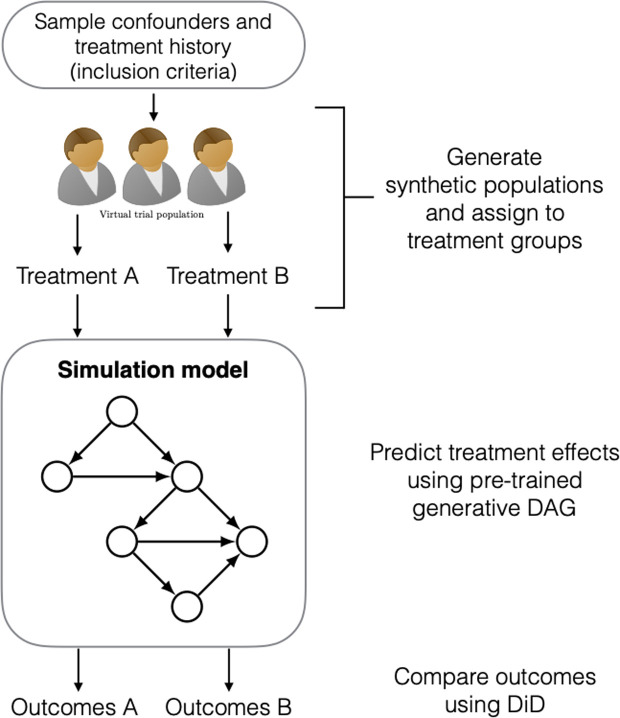
After training, we sample virtual populations according to some known RCT inclusion criteria and use the model to simulate the effect of treatment under different interventions (i.e., emulating the real trial results). We call this process v*irtual trial emulation.*

To overcome these issues, we aimed to develop an emulation model that incorporates the RCT process into a virtual environment, enabling the interpretation of interventions and treatment effects from virtual populations. Two design criteria must be satisfied to achieve this. First, the model requires an understanding of the mechanisms connecting interventions made on the real-world populations to their corresponding effects. Second, the model must be able to produce ‘realistic’ virtual populations, in the sense that they sufficiently represent the variables describing the clinical data of the real-world populations. In this paper, we develop a model that satisfies both requirements by leveraging generative artificial intelligence (AI) algorithms and casual learning. Specifically, we use generative adversarial networks (GANs) [8] to construct a virtual target trial emulation model that learns to generate realistic patient populations for performing targeted trials within a virtual space. This is achieved through an optimization process that minimizes the discrepancy between two sets of distributions: one representing the RWE data, and the other describing the virtual data generated by the GAN (Fig 1 and Fig 2). We modify the original GAN architecture to incorporate the causal relationships amongst the data using directed acyclic graphs (DAG). This enables us to learn the causal effect of treatments from real-world observational data in patient populations. The resulting algorithm constitutes our trial emulation model, and we demonstrate its effectiveness to perform virtual targeted trials by emulating the findings of an established RCT in diabetes research, namely, the LEAD-5 trial (Liraglutide Effect and Action in Diabetes) [9].

Unlike traditional trials, our approach does not require the recruitment of patients. Instead, we apply retrospective eligibility criteria to real-world clinical data in order to emulate an existing trial design (e.g., LEAD-5). This design not only enables validation of our method against a known RCT, but also opens the possibility to simulate trial outcomes in patients who would typically be excluded from randomized studies, such as those with comorbid conditions or polypharmacy. As such, causal AI-powered emulation offers a promising tool for evaluating treatment effects in broader, more clinically representative populations.

The core of the emulation model encodes causal relations among diabetic treatments and patient clinical data into its data generating process, while controlling for known confounding variables (e.g. patients’ demographics, clinical measurements, and treatment history) by setting them to meet trial eligibility criteria. Following the RCT framework, the effect size can then be estimated by randomly allocating the virtual populations to different treatment arms and computing pair-wise differences in outcomes. The virtual populations are sampled from marginal distributions aligned with the LEAD-5 trial population. This sampling approach was chosen to ensure comparability with the original trial design, where treatment groups are assumed to be exchangeable conditional on inclusion criteria, and marginal balance is sufficient to estimate the average treatment effect. We show that our emulation model correctly identifies the reductive effects of liraglutide on HbA1c levels and bodyweight established in LEAD-5, compared to relevant control groups. We also examine the treatment effects in real-world populations, and demonstrate how treatment effects evolve when extending the model to virtual patients who lie outside the original trial criteria, highlighting the capacity of the emulation framework to extrapolate RCT results into more phenotypically diverse T2DM populations. Our emulation model therefore provides a promising framework to extrapolate RCT results into wider, more phenotypically diverse T2DM populations currently out-of-reach in practice, which has strong implications to support treatment decision-making at the patient-level and for broader policy recommendations.

As regulatory bodies increasingly consider the use of AI-generated evidence in decision-making, our emulation framework provides a scalable and rigorous approach to trial replication and extension. By enabling real-time counterfactual exploration and causal effect estimation in silico, this approach can play a pivotal role in post-market surveillance and health technology assessments, particularly in settings where conventional trials are infeasible or ethically constrained. Overall, causal AI models and virtual patient simulations offer a powerful way to supplement traditional approaches to evidence generation, particularly in post-market surveillance and health technology assessment (HTA). In contexts where pragmatic or post-approval trials are logistically challenging or ethically constrained, virtual cohorts based on real-world data can help evaluate treatment effectiveness, explore heterogeneity of response, or simulate long-term outcomes across diverse patient populations. These methods are not intended to replace randomized trials in a foreseeable future, but rather to augment existing evidence by enabling scalable, timely, and reproducible causal analysis.

## Results

Our experiments demonstrated that when patients met the LEAD-5 inclusion criteria (Table 1),

**Table 1:** Patient baseline characteristics from LEAD-5 [9] and SCI-Diabetes.

Variable	LEAD-5	SCI-Diabetes	*p-value*
Age (years)	57.6 (9.5)	68.1 (11.2)	<.0001
Body mass index (kg/m^2^)	30.4 (5.3)	31.3 (4.8)	<.0001
Diastolic blood pressure (mmHg)	80.8 (9.1)	77.6 (9.0)	<.0001
Systolic blood pressure (mmHg)	135.0 (15.0)	134.7 (15.0)	0.033
HbA1c (mmol/mol)	67.2 (7.5)	75.4 (17.7)	<.0001

Values are mean (SD).

Measurements for HbA1c in LEAD-5 were converted from (%) to (mmol/mol) using standard conversion formulas.

p < 0.05 (Mann-Whitney U test) indicates a significant difference between the corresponding features.

### Virtual trial emulations on LEAD-5

We present our main results in Table 2, which summarises the LEAD-5 findings we aimed to emulate, together with the corresponding predictions from our emulation model.

**Table 2:** Comparison in clinical outcomes for T2DM between real (LEAD-5) and Causal AI-powered (Virtual) trials.

Outcome	LEAD-5		Virtual	
*HbA1c*	*Difference: mmol/mol*	*p-value*	*Difference: mmol/mol*	*p-value*
GLP-1 vs glargine	-2.62 (-4.26, -0.87)	0.0015	-1.21 (-1.42, -1.00)	<.001
GLP-1 vs placebo	-11.91 (-13.99, -9.84)	<.0001	-2.58 (-2.78, -2.37)	<.001
*Bodyweight*	***Difference: kg***	***p-value***	***Difference: kg/m*** ^***2***^	***p-value***
GLP-1 vs glargine	-3.43 (-4.00, -2.86)	<.0001	-0.79 (-0.96, -0.63)	<.001
GLP-1 vs placebo	-1.39 (-2.10, -0.69)	0.0001	-0.61 (-0.77, -0.45)	<.001
*SBP*	***Difference: mmHg***	***p-value***	***Difference: mmHg***	***p-value***
GLP-1 vs glargine	-4.51 (-6.82, -2.20)	0.0001	-2.99 (-2.62, -1.89)	<.001
GLP-1 vs placebo^†^	-2.53 (-5.36, 0.29)	0.0791	-2.38 (-2.72, -2.03)	<.001

For Virtual trials, difference-in-differences (DiD) was used to compare intervention groups.

Significant differences (p < 0.05) and 95% confidence intervals were obtained with bootstrapping.

† p > 0.05 indicates no significant difference between treatment groups.

**HbA1c.** LEAD-5 established that Liraglutide reduced HbA1c significantly compared with both glargine (-2.62mmol/mol (−0.24%) difference; p = 0.0015) and placebo (-11.91mmol/mol(−1.09%) difference; p < 0.0001). Through pairwise comparisons with DiD, we found our emulation model predicted GLP-1 to produce a significant HbA1c reduction compared to both basal insulin (-1.21 mmol/mol (-0.11%) difference; p < 0.001), and placebo (-2.58 mmol/mol (-0.24%) difference; p < 0.001). Although the effect sizes are different compared to LEAD-5, our model identifies the correct ranking amongst the treatment arms.

**Bodyweight.** LEAD-5 also demonstrated that Liraglutide produced significantly greater weight loss compared with both glargine (−3.43 kg difference; p < 0.0001) and placebo (–1.39 kg difference; p = 0.0001). We performed a similar analysis using body mass index (BMI) and found that our model provides a similar conclusion: GLP-1 reduced BMI significantly compared to both basal insulin (-0.79 kg/m^2^ difference; p < 0.001) and placebo (-0.61 kg/m^2^ difference; p < 0.001). What was different with our virtual trials, however, was that BMI increased in all treatment arms, instead of reducing bodyweight as in LEAD-5. In our case, GLP-1 produced a significantly smaller weight gain compared with glargine and placebo.

**Systolic blood pressure** LEAD-5 demonstrated a significant reduction in SBP compared with glargine (−4.51 mmHg difference; p = 0.0001), but not with placebo (−2.53 mmHg difference; p = 0.0791). Our simulations suggest GLP-1 reduces SBP significantly more than basal insulin (-2.99 mmHg difference; p < 0.001) and with GLP-1 vs placebo (-2.38 mmHg difference; p < 0.001). Our model’s predictions therefore match the LEAD-5 conclusions for the GLP-1 and basal insulin comparison, but not between GLP-1 and placebo.

### Counterfactual emulations

Given that our model can carry out virtual trials that strongly adhere to LEAD-5, we are now well-positioned to use it to examine our proposed counterfactual scenarios. Our goal here is to demonstrate whether stepping outside of the LEAD-5 criteria and administering alternative treatments to a set of real patients from SCI-Diabetes, we arrive at different conclusions to our emulations from before (Table 2). We present the counterfactual results in Table 3. The columns (GLP-1, Insulin, and Placebo) indicate the drug that patients actually received. The first three rows show the Difference-in-Differences (DiD) comparisons of HbA1c measurements between two treatments within each drug group (column). The next six rows present the corresponding results for BMI and SBP, respectively.

**Table 3:** Difference-in-differences (DiD) for the counterfactual simulations on real patients from SCI diabetes.

Outcome	GLP-1		Insulin		Placebo	
*HbA1c*	*Expected (95% CI)*	*p-value*	*Expected (95% CI)*	*p-value*	*Expected (95% CI)*	*p-value*
GLP-1 vs placebo^†^	-0.32 (-0.62, -0.02)	0.03	0.10 (-0.17, 0.37)	0.472	-3.25 (-3.50, -3.00)	<0.001
GLP-1 vs insulin	0.64 (0.34, 0.94)	<0.001	1.10 (0.83, 1.36)	<0.001	-2.67 (-2.92, 2.42)	<0.001
*BMI*	***Expected*** ***(95% CI)***	***p-value***	***Expected*** ***(95% CI)***	***p-value***	***Expected*** ***(95% CI)***	***p-value***
GLP-1 vs placebo	-0.66 (-0.76, -0.55)	<0.001	-0.65 (-0.74, -0.56)	<0.001	-0.64 (-0.76, -0.53)	<0.001
GLP-1 vs insulin	-0.80 (-0.90, -0.70)	<0.001	-0.83 (-0.93, -0.74)	<0.001	-0.82 (-0.93, -0.70)	<0.001
*SBP*	***Expected*** ***(95% CI)***	***p-value***	***Expected*** ***(95% CI)***	***p-value***	***Expected*** ***(95% CI)***	***p-value***
GLP-1 vs placebo	-2.44 (-2.72, -2.17)	<0.001	-2.64 (-2.94, -2.34)	<0.001	-2.22 (-2.48, -1.96)	<0.001
GLP-1 vs insulin	-2.72 (-2.99, -2.44)	<0.001	-3.00 (-3.30, -2.70)	<0.001	-2.86 (-3.12, -2.59)	<0.001

Results are for HbA1c [mmol/mol], body mass index (BMI) [kg/m^2^], and systolic blood pressure (SBP) [mmHg].

Each column represents the real patient group that we use in our emulation model to predict the effect of three different treatments: placebo, basal insulin, and GLP-1.

Each row is the corresponding DiD for effect size comparisons between these treatments.

† p > 0.05 indicates no significant difference between treatment groups.

Overall, we find that across the real treatment groups, the effect of intervention is most obvious in HbA1c, while BMI and SBP remain relatively unchanged from the previous emulations (Table 2). Two key observations arise in the HbA1c comparisons: (1) when we treat SCI-Diabetes patients belonging to the GLP-1 group with basal insulin (Table 3, GLP-1 column), we find that GLP-1 is significantly less effective for HbA1c reduction (GLP-1 vs insulin: + 0.64 mmol/mol difference; p < 0.001); (2) patients belonging to the real basal insulin group (Table 3, Insulin column) benefit more from both placebo (GLP-1 vs placebo: + 0.10 mmol/mol difference) and basal insulin (GLP-1 vs insulin: + 1.10 mmol/mol difference; p < 0.001) than GLP-1, in terms of lower HbA1c, though the result with placebo is not significant (p = 0.472). The implications of these findings are intriguing, but hardly surprising: GLP-1 is not always the most effective treatment. Recall that for these experiments, we operate within significantly different initial conditions to those specified by LEAD-5, particularly with HbA1c (Table 1). The benefits of GLP-1 are therefore only clear when we intervene on phenotypically narrow cohorts: when we relax these conditions (as we do here), the conclusions are free to change. The result is not unique in of itself. The novelty is that it was discovered automatically by our emulation model, based on the causal structure learned from RWE data.

## Discussion

How RCT results translate into real-world, *phenotypically diverse* populations remains an open question. To make a first step in this direction, we developed a novel emulation model to perform clinical trials on patients within a virtual space.

According to the experimental results, the virtual trial emulations produced the same ranking between the three treatment arms in LEAD-5 [9], suggesting that GLP-1 produces more favourable patient outcomes in terms of HbA1c, BMI, and SBP reduction (Table 2). However, the counterfactual experiments with real patients who did not fall into the baseline characteristics of LEAD-5 presented different performance rankings between the drugs (Table 3). For example, amongst patients who received basal insulin in practice, the simulations revealed GLP-1 to be significantly less effective than basal insulin for reducing HbA1c, suggesting that LEAD-5 trial outcomes cannot be simply extrapolated to cover other patient populations. Our emulation model therefore has the potential to provide useful evidence for examining the generalizability of RCT results for real-world clinical practice.

Overall, our emulations closely replicated the conclusions of LEAD-5 for HbA1c and BMI. However, we observed a discrepancy in the effect of GLP-1 vs placebo on SBP: where LEAD-5 found no significant difference, our emulation suggested otherwise. The specific effect size among individual drugs was also exaggerated in most cases. We believe that the following differences between the real (LEAD-5) and casual AI-powered (Virtual) trial emulations were responsible for these discrepancies:

- The SCI-Diabetes dataset presents the key drug of interest as GLP-1*,* which is more general than the specific drug (i.e. Liraglutide) tested in LEAD-5. Hence the training of the emulation model, and the testing with the trial emulations, have aggregated the mean effect of several GLP-1 drugs within a single drug category. Moreover, both basal insulin and placebo are involved as drug categories in the emulations, which are not identical to the corresponding drugs involved in the original trials in LEAD-5 (i.e., insulin glargine and liraglutide placebo, respectively).
- The SCI-Diabetes dataset also considered different measurement units to LEAD-5 for HbA1c and bodyweight; LEAD-5 considered HbA1c with [%] and bodyweight [kg], whereas SCI-Diabetes considered [mmol/mol] and [kg/m^2^], respectively. Notice also that we measured changes in BMI rather than absolute bodyweight. This was due to a limitation in the SCI-Diabetes dataset, as it does not include individual weight measurements, which were the primary endpoint used in LEAD-5 for assessing weight-related outcomes. Instead, we used BMI (kg/m²) as a proxy, acknowledging that it may respond differently to treatment than absolute weight. Nonetheless, the relative treatment effects were preserved, with GLP-1 showing more favourable outcomes than basal insulin and placebo, consistent with the directional findings of LEAD-5.
- Although the emulations have tried to re-create the LEAD-5 trial populations (Table 1), it is not possible to replicate a completely identical cohort due to different settings between the trial and real-world clinical practice.

We acknowledge several limitations in our technological and data pre-processing methodology:

- For the trial emulations, we sampled virtual patients from the emulation model using a random-based approach to generate sample patients according to the inclusion criteria. A more complete and principled generation approach would probably be to instead consider relationships amongst the pre-features during the sampling.
- We acknowledge that sampling covariates independently may overlook the real-world correlations among variables, potentially undermining ecological validity. In future work, we plan to integrate causal-preserving generative models or Bayesian bootstrapping to retain joint dependencies while sampling. Such approaches would allow us to preserve the structure of the real-world joint distribution, improving the realism of virtual patients.
- Our pre- and post-treatment features were based on yearly median values, which may have obscured fine-grained treatment effects and introduced errors into the learning process.
- Our emulation assumes causal sufficiency, where all relevant confounders have been measured. This assumption is partially supported by our alignment with the LEAD-5 trial design, which guided our selection of covariates. By choosing variables deemed essential in a real randomized setting, we aim to mitigate the risk of omitted confounding. In addition, our approach to trial emulation does not estimate causal effects directly from observational data. Instead, we first learn a structural causal model (SCM) from real-world data, incorporating covariates that align with those used in the LEAD-5 randomized trial. This alignment ensures that key confounders and effect modifiers are included, based on an established trial design, and is intended to block backdoor paths in the treatment–outcome relationship. We then use a generative model to simulate virtual patient populations that satisfy the LEAD-5 eligibility criteria, and assign treatments randomly within these synthetic cohorts. This process emulates the conditions of a randomized trial, thereby eliminating confounding by design during the effect estimation stage. However, we acknowledge that the assumption of no hidden confounding applies to the model training phase, where causal structure and parameters are learned from observational data. As with any real-world dataset, the possibility of unmeasured confounders remains a limitation at this stage. Future work could address this by integrating latent confounder modelling or conducting sensitivity analyses to evaluate the robustness of the learned structure.

Overall, we showed that our algorithm successfully emulated the main conclusions of a real RCT in diabetes research, namely, the LEAD-5 trial [9]. We then used our algorithm to investigate counterfactual treatment scenarios that extrapolate treatment effects to populations out with LEAD-5. We discovered that the main finding of LEAD-5 did not translate into phenotypically diverse populations, where the key investigative drug (Liraglutide) was not the most effective choice in all patient cases. Our algorithm therefore has the potential to inform medical professionals about the effectiveness of drugs at the individual patient-level, and as an advisory tool for examining current clinical guidelines. Future work will investigate our algorithm within other specialties (e.g., oncology), and explore strategies to deal with our study’s various shortcomings (e.g., closer matching between real and virtual drugs, and missing data).

While our work is framed around type 2 diabetes and the LEAD-5 trial, the causal AI-powered trial emulation framework we propose is generalisable across therapeutic domains. The core components of our approach, learning structural causal models from real-world data, generating synthetic cohorts that meet trial criteria, and estimating treatment effects under randomized assignment, are applicable in any setting where robust observational data is available and randomized trials are limited or incomplete.

For example, in cardiology, GLP-1 receptor agonists have been investigated for heart failure and cardiovascular risk reduction, but trials often include selective populations and lack long-term real-world evidence. In oncology, the increasing use of targeted therapies, often approved on the basis of small, stratified trials, creates a strong demand for external validation and post-approval generalisability studies. Similarly, in rare diseases, the small number of eligible patients makes full-scale RCTs challenging or infeasible. Another pressing application is in the context of **multimorbidity**, which is the co-existence of two or more chronic conditions within an individual. Multimorbidity is associated with high mortality, reduced quality of life, and significant treatment burden, yet it remains underrepresented in most RCTs due to strict exclusion criteria. For instance, patients with multimorbidity in diabetes or heart failure are frequently excluded from trials, creating a large evidence gap. As a result, clinical guidelines tend to be disease-specific and may recommend treatments that interact negatively or impose conflicting burdens on patients with complex health profiles.

In these contexts, trial emulations can supplement limited trial data, generate synthetic comparators, and support regulatory and health technology assessment processes. By enabling counterfactual reasoning and treatment effect estimation in synthetic populations, our method provides a flexible and scalable framework to explore efficacy, safety, and heterogeneity of treatment effects beyond diabetes, potentially accelerating evidence generation in a wide range of clinical areas.

## Materials and methods

### Ethics statement

This study (ID: GSH22DI002) was approved by the Research and Innovation Peer Review Committee and the Local Privacy Advisory Committee at NHS Greater Glasgow and Clyde, UK, together with the Safe Haven platform team that provided access to the retrospective data [10]. This included both Research and Innovation Management and Research Ethics Committee approval.

We sought to emulate the findings of the LEAD-5 trial within our virtual trial populations. The goal in LEAD-5 was to confirm whether liraglutide, a glucagon-like peptide-1 (GLP-1) receptor agonist, caused a greater reduction in HbA1c and bodyweight compared to insulin glargine and placebo in people with T2DM. To estimate the effect of treatment within our virtual environment, the virtual populations were first generated according to the trial inclusion criteria of LEAD-5, and then randomly assigned to one of three LEAD-5 treatment arms: placebo, basal insulin (glargine), or GLP-1 (liraglutide). We used proton pump inhibitors (PPIs) or statins as our inactive comparators. Following LEAD-5, each arm was in combination with metformin and sulfonylurea.

In alignment with the LEAD-5 trial, where a placebo was used as an inactive control, we selected proton pump inhibitors (PPIs) and statins as inactive comparators. These medications are commonly prescribed in the T2DM population but are not known to directly affect glycemic outcomes such as HbA1c. Their inclusion helps provide a pragmatic approximation of placebo-like controls in real-world observational data.

### Background in causal learning

In a randomised controlled trial, randomization ensures that the treatment and control groups are similar, avoiding confounding effects. In observational studies, however, individuals are not randomly assigned to treatments, meaning we often face confounding variables (or confounders) that influence both the treatment and the outcome. To adjust for confounding, and therefore emulate the conditions of randomised trials from real-world observational data, we use techniques from causal learning [14]. The principles of causality have been established through foundational work by researchers like Judea Pearl, who developed frameworks and formalised causal reasoning with causal graphs. These frameworks enable us to distinguish between correlation and causation, use diagrams to specify causal assumptions, and estimate effects as if a randomized intervention had been conducted.

In real-world data, we often find correlations or associations between variables, but these do not necessarily imply a causal relationship. This distinction is crucial in causal inference, where the goal is to understand the direction and nature of causal influences, not just statistical associations. Pearl’s work [14], particularly in establishing *d-separation* for determining conditional independence, and the *do-operator* for simulating interventions, laid the groundwork for causal learning in observational data. These concepts help us replicate the effects of random assignment by allowing us to virtually “intervene” in a dataset, thus enabling robust causal analysis without requiring a traditional experimental design.

To emulate this control in observational data, we use the *do-operator* to simulate an intervention. The *do-operator* sets the treatment variable *T* to a specific value (e.g., *T = 1* for treated) and “breaks” the usual dependencies in the data that would naturally influence T. By applying *do(T = 1)* or *do(T = 0)* we simulate the effect of assigning individuals to treatment or control groups as we would in a randomized trial. This approach allows us to calculate the interventional distribution of the outcome, which is akin to observing outcomes in different treatment arms.

A Directed Acyclic Graph (DAG) plays a crucial role in applying the do-operator in causal inference because it visually represents the causal relationships among variables in a system and helps clarify the assumptions needed to isolate causal effects. In a DAG, each node represents a variable, and directed edges (arrows) between nodes represent causal effects. Importantly, DAGs must be acyclic, meaning there are no feedback loops. By constructing a DAG, we map out the relationships between treatment, outcome, and potential confounders. To isolate the causal effect of treatment on the outcome, we “block” any backdoor paths from the treatment to the outcome. This is enforced through *d-Separation*, a criterion used on DAGs to assess whether a set of variables effectively “blocks” paths between other variables, helping to determine conditional independence. If two nodes are d-separated, it means that conditioning on certain variables can block the influence or “flow” of information between them. This property helps us decide which variables to condition on when aiming to isolate causal effects. Using d-separation on the DAG, we identify confounders (common causes of treatment and outcome) that must be controlled. For instance, if we observe that a variable Z influences both treatment T and outcome Y, conditioning on Z (adjusting for it in our analysis) allows us to control this confounding effect. This adjustment mimics the balance achieved in randomized trials, making treatment and control groups comparable.

DAGs are often formalised through structural causal models (SCMs), which are mathematical frameworks that specify causal relationships between variables. Structural equations in the DAG allow us to specify how each variable causally depends on others. For the outcome Y, we create an equation that accounts for both the treatment T and any other relevant predictors or confounders. These structural equations enable us to simulate or predict outcomes under different treatment scenarios, emulating what we would see if we could control treatment assignments.

Once we’ve controlled for confounding, we can estimate the potential outcomes for each individual: Y(1) if treated and Y(0) if not treated. In observational data, we only see one of these for each individual, but SCMs and adjustments allow us to estimate the missing counterfactual outcome. By calculating these potential outcomes for each individual in the dataset, we then compute the Average Treatment Effect (ATE):


ATE=E[Y(1) −Y(0)]\] (1)

which serves as the average causal effect analogous to the treatment effect measured in a randomised trial.

### The emulation model

We recognise that observational datasets are subject to various biases, including selection bias and confounding. To mitigate these, we employed a causal structure learning approach to adjust for observed confounders and simulate treatment assignment under randomized conditions. Our model was designed to isolate causal effects by controlling for known covariates and enforcing d-separation in the DAG.

By following the steps below, we create conditions that resemble those of a randomised trial, enabling the estimation of causal effects from observational data:

- Train the emulation model with the data to learn a DAG representing the causal structure, along with the associated structural equations that define treatment-outcome relationships.
- Use d-separation to identify confounding variables and block backdoor paths in the DAG, ensuring comparability between treatment and control groups.
- Use the emulation model to generate virtual cohorts for both treatment and control arms by setting the confounding variables (e.g., patient demographics, clinical measurements, and treatment history) to meet the trial eligibility.
- Calculate potential treatment outcomes and ATE for both treatment and control groups using the emulation model.
- Estimate Difference-in-Differences (DiD) for pairwise comparisons between different arms.

In addition to following the standard causal inference framework, we designed our emulation model to unify recent developments in machine learning-based generative modelling and causal structure (DAG) learning within a single framework. Specifically, we combined the Wasserstein GAN architecture (WGAN-GP) [11] with the NOTEARS-MLP method [12]. By involving the causal dependencies between the data variables from NOTEARS-MLP within the WGAN-GP architecture, we learn how to simulate the true generative process underpinning the real-world data. This is achieved through an extensive training process that solves a distance minimisation problem between the real and generated data (Fig 1). During training, the emulation model learns to represent the causal structure as a directed acyclic graph (DAG), which maps out the treatment effect while adjusting for known confounders (i.e., common causes, such as age and medical history) that influence both treatment assignment and outcomes. This understanding is crucial for virtual trial emulation: by disentangling the direct and indirect influences of these confounders, we enable more accurate estimation of treatment effects across diverse populations. The resulting framework allows us to generate synthetic (virtual) patients from the causal mechanisms among treatments, patient data, and known confounders to simulate the effect of treatment in a purely virtual manner (Fig 2).

### Model training and hyper parameters tunning

Our model involves training a generative adversarial network (GAN) under an augmented Lagrangian optimization framework, which incorporates a structural constraint to enforce acyclicity in the learned causal graph. This setup introduces a set of hyperparameters across three components: the GAN architecture, the augmented Lagrangian process, and the training schedule. The learning rates for both the generator and discriminator were initialized at 3 × 10^−4^, and we employed a cosine annealing schedule to adjust the learning rate dynamically throughout training. In this schedule, the learning rate follows a cosine curve, starting high and gradually decreasing to a minimum (in our case, 7 × 10^−6^) over each training cycle. This gradual, non-linear decay helps the model avoid premature convergence to suboptimal solutions and improves generalisation by allowing the model to take large steps early in training and smaller, more precise steps as it approaches convergence. Importantly, we used warm restarts at regular intervals (every 300 epochs), resetting the learning rate to its initial value at the beginning of each new cycle. These restarts encourage exploration of new regions of the parameter space and reduce the risk of the model becoming stuck in a poor local minimum.

In addition to optimizing predictive accuracy, our model was designed to learn an interpretable causal structure. To ensure the learned graph remained a directed acyclic graph**,** we used an augmented Lagrangian optimization framework. This approach incorporates the DAG constraint as a soft penalty into the model’s loss function, enabling simultaneous optimization of both fit and structural validity. The penalty is governed by a Lagrangian multiplier that increases over time, making the constraint progressively stricter as training progresses. Specifically, at the end of every epoch cycle, we updated the Lagrangian multiplier and restarted the learning rate schedule, thereby synchronising constraint enforcement with the model’s ability to explore new parameter configurations. This coupling of constraint tightening and learning rate restart was found to significantly improve the convergence of the model toward valid causal graphs.

To assess convergence and training quality, we monitored several diagnostic metrics. For adversarial training stability, we tracked the Wasserstein loss and gradient penalty. To evaluate how closely the generated synthetic data matched the real data distribution, we computed both the maximum mean discrepancy (MMD) and the mean squared error (MSE) between real and synthetic observations. These diagnostics ensured that the model not only learned a plausible causal structure but also generated realistic virtual patient data suitable for downstream trial emulation.

### Dataset and pre-processing

We prepared a training dataset for our emulation model that reflected clear causal signals between the interventions and clinical outcomes. For training, we used the SCI-Diabetes dataset hosted on the Glasgow Safe Haven platform [10], which comprises an inclusive regional cohort of individuals with diabetes containing a broad range of longitudinal demographic, phenotypic, biochemical and screening data. There are approximately 300K individuals with diabetes, where 3K individuals with MODY (Maturity-onset diabetes of the young) are recorded with certainty (genetic information) along with records of individuals with negative genetic test results. The dataset consists of an array of variables for clinical measurements, drug prescriptions, and demographics for 56,476 unique patients with T2DM. We selected a subset of these variables according to the LEAD-5 outcomes, specifically, plasma glucose (HbA1c: mmol/mol), body mass index (BMI: kg/m^2^), and systolic blood pressure (SBP: mmHg), in addition to other relevant features such as urine albumin-to-creatinine (UAC) ratio, estimated glomerular filtration rate (eGFR), cholesterol, and creatinine levels. Table 1 provides an overview of the clinical variables and patient characteristics relevant to LEAD-5.

To preserve the causal validity of the structural model, we removed all records with missing data in any of the variables required for model training. This complete-case analysis ensures that causal dependencies are learned from fully observed instances, thereby avoiding biases that may arise from imputation methods, which can distort the underlying data-generating mechanisms, particularly in high-stakes clinical settings such as diabetes care. While this decision helps maintain the internal consistency of the learned causal graph, it comes at the cost of a reduced training set. We acknowledge this as a limitation; however, we verified that the marginal distributions of key clinical variables remained stable after excluding incomplete records, suggesting that the removal of missing data did not meaningfully alter the underlying population characteristics. We therefore consider the preserved dataset sufficient for training the structural model and conducting virtual trial emulation across the three main comparators relevant to the LEAD-5 replication.

To train our emulation model, we prepared the dataset to represent each patient’s full treatment record as a series of pre- and post-treatment measurements collected over time. Each new treatment marks a distinct point in time, allowing us to distinguish clear responses to individual treatments and to account for all drugs administered up to that point. The result is a set of variables to represent treatment history, each encoded as a binary indicator for their presence (1) or absence (0). For the rest of the paper, we refer to the pre- and post-treatment clinical features as pre- and post-features, respectively. Given a distinct treatment and associated timestamp, pre-features were collected from 9 months prior to intervention, and post-features within a subsequent period of 12 months. If multiple measurements were collected during these periods, we used their median values.

**Fig 3:**
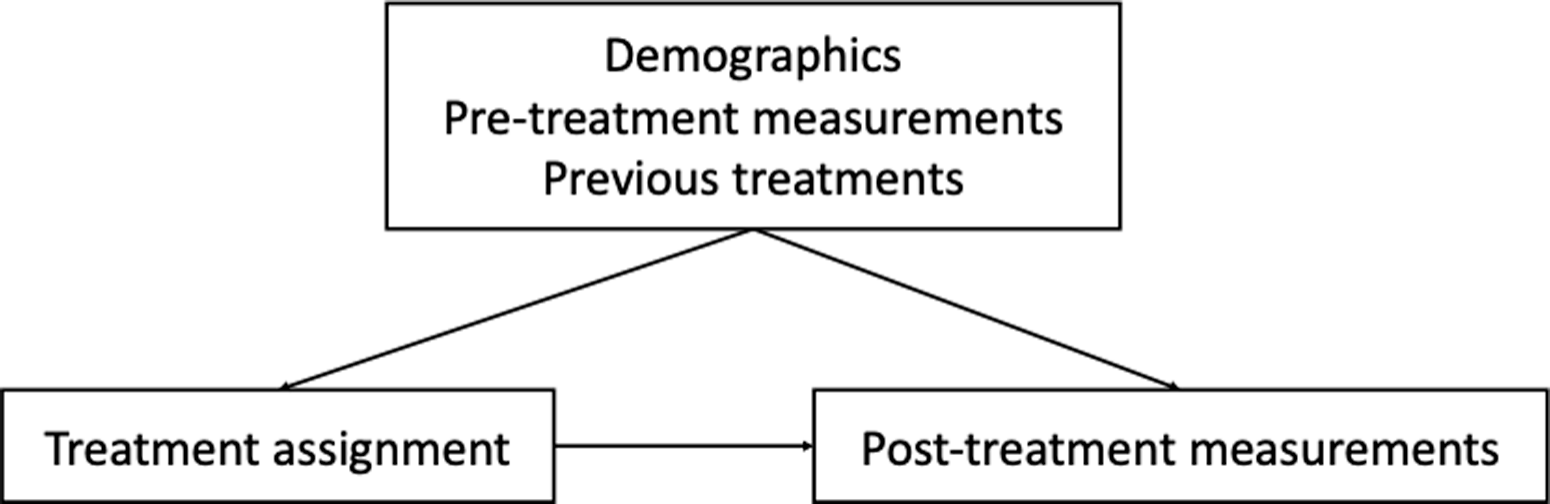
Overall causal structure between treatment, post measurements and confounders.

We divide the variables into three main categories, including treatments, post-treatment measurements and the confounders that involve patient demographics, pre-treatment measurements and prior-treatments. The overall causal structure is shown in Fig 3. In addition to the direct causal link between treatment and post-treatment measurements, we account for confounding effects from demographics, pre-treatment measurements and previous treatment to the treatment assignments and post-treatment measurements. Through learning from the data distribution, we infer the exact causal graph structure between the variables, together with the structural equations that are associated to the graph to enable synthetic data generation for trial emulation.

Most of the confounders and post-treatment measurements are continuous variables. We consider each drug as a separate discrete variable. To reduce the number of possible drug combinations, and to account for the confounding effects imposed by other prescribed drugs, we categorise all drugs into their corresponding classes. This allows us to model the ‘global’ causal effects of each drug, under the assumption that all drugs within each class cause similar effects to a given patients’ features. Thus, the treatment (drug) nodes are represented by binary variables. Each drug is associated with one designated binary node. The node is set to 1 if the associated drug is applied to patients in the treatment, and 0 otherwise.

### Virtual trial emulations

Given the trained emulation model, we performed virtual trial emulations by generating a cohort of virtual patients that satisfied the LEAD-5 inclusion criteria (Table 1). We assume independent normal distributions for the demographic variable and pre-features and study the effect in the primary outcome of LEAD-5, namely, changes in starting HbA1c. We also examine the effects on bodyweight (Note: BMI rather than absolute weight) and systolic blood pressure (SBP), which form the secondary outcomes of LEAD-5. To match the group sizes in LEAD-5, we randomly assigned n = 232 virtual patients to one of our three intervention groups (i.e., placebo, glargine, or GLP-1). As with treatment assignment in an RCT, our sampling ensures that we eliminate any confounding and thus prevent bias in the estimation of the drug effects (since all virtual patients have similar conditions prior to treatment), enabling meaningful group comparisons. Once the pre-treatment and drug variables are assigned, the model calculates the post-treatment measurements of the virtual patients, yielding full records of the virtual patients in each group. The trial results are computed as the mean and standard deviation over 60 random seeds. We used the difference-in-differences (DiD) method [13] to quantify pairwise comparisons in outcomes between each group.

Our primary goal in sampling was to match the LEAD-5 inclusion criteria, which define marginal constraints over demographic and clinical features. For this reason, we used independent normal distributions calibrated to the LEAD-5 population-level statistics, ensuring marginal consistency.

### Counterfactual emulations

We conducted a complementary study based on counterfactual emulations, which examine clinical questions about hypothetical treatment scenarios. For example: *“Would this patient have responded more favourably to a different treatment?”*. In other words, we compute the post-features in response to treatments different to those prescribed in practice. We specifically emulate counterfactual treatments where different drugs are applied to the same real patients from our observational dataset (SCI-Diabetes). In our experiments, we identified patients in SCI-Diabetes according to the drugs they received in practice. For each real patient, the virtual trial proceeds by administering each of our three treatments and computing the pairwise DiD between each group. Importantly, recall the statistically significant differences between the SCI-Diabetes and LEAD-5 features (Table 1), which imply that we should expect changes in effect size between the SCI-Diabetes patient cohorts compared to those satisfying the LEAD-5 criteria.

### Measurement of synthetic data distribution

To assess the realism of the synthetic populations used in our virtual trials (Table 2), we compared them to the subset of real patients from the observational dataset who met the LEAD-5 trial inclusion criteria. We computed distances between real and synthetic data distributions for key baseline variables including age, HbA1c, BMI, and systolic blood pressure. In Supplementary Table S, we report means, standard deviations, Maximum Mean Discrepancy (MMD)**,** and Wasserstein distance for each covariate. MMD is a non-parametric kernel-based measure that quantifies the difference between two probability distributions; lower values indicate higher similarity. Wasserstein distance, also known as Earth Mover’s Distance (EMD), measures the minimal cost required to transform one distribution into another, making it especially sensitive to differences in distributional shape and support. Together, these metrics provide complementary perspectives on how closely the synthetic data aligns with the real data distributions. Further, these observations are illustrated in Fig S, which presents overlaid density plots for each post-treatment variable.

Overall, the synthetic data replicates the central tendencies of the real-world distributions well, as reflected by low MMD scores across all variables and modest Wasserstein distances. As shown in Fig S, variables such as creatinine, eGFR**,** and urinary albumin concentration (UACR) show particularly close alignment (i.e. the synthetic and real distributions for these features overlap well, including both the peak and the distribution tails), which suggests that the generative model captures key renal function markers effectively. However, some divergence is observed in variables with greater biological variability, such as HbA1c, BMI, and blood pressure (SBP/DBP). In these cases, the synthetic data appears more sharply peaked and less dispersed than the real data, indicating an underrepresentation of the natural variation and extremes found in real populations. This trend aligns with slightly elevated Wasserstein distances in Table S for these variables (e.g., 8.29 for HbA1c and 1.57 for BMI). These discrepancies likely stem from the model’s tendency to regularise around typical values, possibly limiting its capacity to capture outlier cases. Despite these limitations, the relative ordering of treatment effects is preserved, and the synthetic data falls within plausible physiological ranges for all variables examined. The combination of distributional metrics and visual comparison supports the conclusion that the generative model produces realistic and interpretable post-treatment outcomes, suitable for downstream applications such as virtual trial emulation and treatment comparison under varied clinical profiles.

## Supporting information

Fig S Density Plots Comparing Real and Synthetic Distributions: Each plot shows the kernel density estimation (KDE) of a clinical variable in the real dataset (blue) and the corresponding synthetic distribution (orange) generated by the model. Variables include (from top left) BMI, cholesterol, creatinine, blood pressure, HbA1c, eGFR, and urinary albumin concentration. Alignment in shape and spread indicates fidelity of synthetic data generation.
(TIFF)

Table S Summary of Real vs Synthetic Data Similarity for Post-Treatment Outcomes. (DOCX)
