# Supplementary figures and images for: Emulating real-world GLP-1 efficacy in type 2 diabetes through causal learning and virtual patients
Source: PLOS Digit Health. 2025 Jul 21;4(7):e0000927. doi: 10.1371/journal.pdig.0000927 (PMC12279107; doi:10.1371/journal.pdig.0000927)

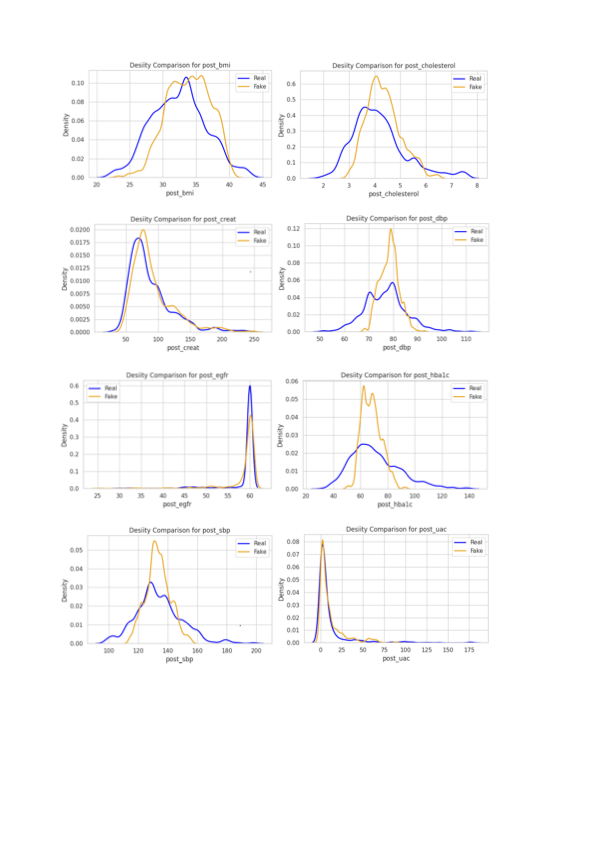

Supplement: Fig S — Density Plots Comparing Real and Synthetic Distributions: Each plot shows the kernel density estimation (KDE) of a clinical variable in the real dataset (blue) and the corresponding synthetic distribution (orange) generated by the model. Variables include (from top left) BMI, cholesterol, creatinine, blood pressure, HbA1c, eGFR, and urinary albumin concentration. Alignment in shape and spread indicates fidelity of synthetic data generation. (TIFF) [file pdig.0000927.s001.tiff]
