# Supplementary material for: Emulating real-world GLP-1 efficacy in type 2 diabetes through causal learning and virtual patients
Source: PLOS Digit Health. 2025 Jul 21;4(7):e0000927. doi: 10.1371/journal.pdig.0000927 (PMC12279107; doi:10.1371/journal.pdig.0000927)
Supplement: Table S — (DOCX) [file pdig.0000927.s002.docx]

| Table S: Summary of Real vs Synthetic Data Similarity for Post-Treatment Outcomes | | | | |
| --- | --- | --- | --- | --- |
| Variable | **Real Mean (SD)** | **Synthetic Mean (SD)** | **MMD** | **Wasserstein** |
| Body mass index (kg/m^2^) | 32.4 (4.1) | 33.8 (3.2) | 0.020 | 1.57 |
| Total Cholesterol (mmol/l) | 4.2 (1.1) | 4.4 (0.7) | 0.059 | 0.36 |
| Creatinine (µmol/l) | 87.8 (34.7) | 92.2 (33.9) | 0.005 | 4.50 |
| Diastolic blood pre (mmHg) | 76.9 (8.7) | 78.4 (3.9) | 0.038 | 3.86 |
| Systolic blood pre (mmHg) | 133.9 (15.6) | 133.5 (8.2) | 0.016 | 5.50 |
| UACR (Urine albumin-to-creatinine ratio) (mg/mmol) | 10.8 (22.5) | 11.3 (15.5) | 0.021 | 3.89 |
| HbA1c (mmol/mol) | 70.8 (18.2) | 68.2 (7.2) | 0.025 | 8.29 |
| eGRF (Estimated Glomerular Filtration Rate) (ml/min) | 55.7 (8.7) | 55.9 (8.2) | 0.065 | 0.90 |
